# The Surgical Treatment of General Peritonitis Due to the Dissemination of Septic Products

**Published:** 1895-02

**Authors:** Oscar H. Allis

**Affiliations:** Philadelphia


### THE SURGICAL TREATMENT OF GENERAL PERITONITIS DUE TO THE DISSEMINATION OF SEPTIC PRODUCTS. *

BY OSCAR H. ALLIS, M.D.,

PHILADELPHIA.

With the confident assurance that a patient has general peritonitis of septic origin, the surgeon of to-day does not hesitate to open the abdominal cavity, repair, if possible, the origin of the disease, and follow this with a thorough flushing of the infected region with properly tempered water; and with what result? Death so promptly and inevitably as to discourage both surgeon and physician from ever attempting it again. Yet such cases are purely surgical, and if any succor is ever to come to them it must be through open surgical measures. These two considerations have alone prompted me to bring this subject before the Academy to-night, and, though I have nothing hopeful to offer in the way of practical experience, I

*Read before the Philadelphia Academy of Surgery, October, 1894.

hope in a comparison of this affection with kindred diseases to offer at least a suggestion for a future course of procedure.

At the outset, let me repeat that I shall have nothing to say upon the origin of the disease or the treatment of the primary lesion; my topic is the treatment of general septic peritonitis, from whatever cause it may arise.

First, is there anything in the nature of the peritoneal membrane itself that makes it so fatal? If we compare the diseases of the thorax with those of the abdomen, the question can be answered in the negative. The pleura is originally a part of the general serous membrane, and, of course, identical with that of the abdomen. Purulent pleurisies are treated to-day with great success. What are the conditions present, and on what does the success depend? The conditions present are the covering of the entire lung and thoracic cavity with a foul, poisonous exudate, and the pouring out into the thoracic cavity of a putrid, offensive, purulent fluid. The accumulation is accompanied with pain, fever from the absorption of septic products, difficulty of breathing from mechanical interference, loss of appetite and emaciation, and, if unrelieved, death. The relief of such a case is simple, and should be carefully inquired into. In its simplest form the treatment consists of free, dependent, unobstructed drainage. Under such treatment so many have gotten perfectly well, that one must seriously ask if water is used to wash out the cavity, of what use it is? Water used upon an indolent leg-ulcer may wash away the effusive accumulations; but it does not, surgically speaking, clean the ulcer. Water medicated, as a strong mercurial solution, does not surgically cleanse the ulcer. It may destroy the superficial layers of the diseased germ-bearing tissue, and render a necessity for dressings less frequent, but to cleanse away the diseased semi-organized tissue the knife must be used and the whole cut away, or the curette employed and the surface scraped until a healthy base is reached. Then, and only then, have we removed the foul covering of the ulcer and obtained a surgically clean surface as a basis of repair.

When we use a simple or medicated fluid in the thoracic cavity we wash away, if the opening be sufficiently free and the stream ample, the dead effete products of the pseudo-membrane that lines the thoracic cavity and covers the lung. The washing does not purify, surgically speaking, the exudate, and even if we employ the so-called germicidal fluids, effect upon the pseudo-membrane is only upon its surface. It may, as in the case of the leg-ulcer, diminish the quantity of pus and the frequency of dressing, but it cannot wholly eradicate or destroy the exudate.

Seeing then, that recoveries take place in purulent pleurisies from drainage without washings, as well as from draining with washings, the conclusion must inevitably follow that *the essential feature of that treatment is drainage.*

But we must not omit a further inquiry into the case, namely, the nature of the pus cavity, for that it really is. The answer to this question is, I believe, the key to the success of treatment. In some cases the pus chamber is a simple cavity. The lung has shrunken and is far removed from the surface of the ribs. In this class there is no opposition of visceral surfaces, and no hindrance to drainage, and if the openings are dependent and ample, the treatment will be unattended with relapse or annoyance. In other cases, owing possibly to previous pleurisies, there are pockets of pus, and imperfect drainage, at best, is obtained. In such cases the dead elements of the exudate are retained in pockets, and the result is imperfect, unsatisfactory response to treatment, and death, though delayed, finally ends the scene.

Let us now take up the conditions present in a purulent peritonitis. My subject demands that I treat this condition when it is full-blown and general; but before doing so I will delay a moment to show the analogy between a local peritonitis due to septic products and the condition present in a purulent pleurisy. In both a serous membrane is involved; in both the abscess cavity is walled off with a pseudo-membrane; in both the recovery is chronic, and can only take place by the slow removal of the exudate and its replacement with healthy granulations, and in both drainage, free and unobstructed, is an uncompromising essential. The success of the operation for the relief of local purulent appendicitis depends not on washing out the cavity with antiseptic lotions, *but in preserving in its integrity the wall of lymph* that has been formed to limit the progress of the disease and prevent septic material from infecting the general peritoneal cavity. So long as a pus chamber can be kept patulous, and the elements of decomposing pus and tissue readily removed, the progress of such cases is to health.

Now what are the conditions in a general peritonitis? Not one, but scores of pockets of poisonous matter—pockets everywhere—with lymph covering every *tissue* of the abdomen. To remedy this the surgeon enters with his hand, and carries a clean warm fluid into every part of the cavity. He breaks open a great number of pus chambers, and removes the ptomaïnes and leucomaïnes that are doing their deadly work. So far very well; but this to be effectual must be repeated. The washing does not purify the exudate—more effete products will cast off and must be gotten rid of. Some means must be taken to prevent adhesions taking place, while at the same the elements of putrefaction and necrotic waste must be removed. Whether drainage-tubes passed to the most dependent parts of the cavity, as a means of continuous flushing, would prevent reattachments, can only be learned by trial. Such a course will be of no real value if partial or trifling. The waste must be driven off, and the stream kept up constantly night and day, until the normal surfaces have regained their tone, and thrown off the exudate. Under a continuous system of flushing or irrigation, the waste products would be made to float constantly to the surface, and be more effectually carried off than by dependent dosal drainage.

Such a course may commend itself to some surgeons, and as it has already been recommended in whole or in part, it will no doubt ere long receive a fair intelligent trial. To my mind the following seems more in keeping with Nature's own plan:

As drainage is the basis of recovery in the allied conditions referred to, I cannot conceive of any adequate dependent drainage except a long median incision—kept open by proper packing—*with the patient prone,* except at the times of dressing. To prevent the sides of the incision from closing, or the intestines from uniting to the lips of the incision, rubber dam thoroughly covered with cerate could be tucked between the abdominal wall and the intestines on each side, with one border emerging from the incision. At the same time, to retard, if not prevent adhesions of the contiguous coils of intestines, the whole serous surface could be well covered with a lubricant-like cosmoline or cerate. If the abdomen has been previously flushed with water, the cavity should be dried before application of the cerate. Were it not that the adhesions tend constantly to form, I should be sanguine that the *prone* position alone, maintained constantly for weeks, would result in recovery. To guard against these, it would seem wise to re-enter the cavity on the following day, and reapply cerate to the walls of the cavity and the entire surfaces of its contents, redressing with rubber dam as before, again placing the patient prone. With dependent drainage, with an ample wound kept patulous, all water, save such as is necessary to preserve outward cleanliness, could be dispensed with. A difficulty might be experienced in keeping the patient in the prone position. This could be accomplished by means of a thin, well-padded strip of wood two or three feet long, strapped transversely to the pelvis.

Although this is the purest theory, it is theory based on successful treatment of allied affections. It is treatment of an extensive pus cavity, with adequate dependent drainage. Drainage from the rectum or vagina may be serviceable when a local pelvic peritonitis is under consideration, but when general, nothing short of a long six-inch median abdominal incision will be adequate for the vast area of disease, and this only can do the work when the incision is freely patulous and made dependent by the persistent prone posture.

In closing, I wish again to call the attention of surgeons to the statement made in the beginning, that the disease under consideration is of a purely surgical character, and that the responsibility in a great measure of its successful management rests with them. Nothing more weighty, nothing more important demands their attention.

